# Supplementary material for: Mechanism of bisphosphonate-related osteonecrosis of the jaw (BRONJ) revealed by targeted removal of legacy bisphosphonate from jawbone using competing inert hydroxymethylene diphosphonate
Source: eLife. 2022 Aug 26;11:e76207. doi: 10.7554/eLife.76207 (PMC9489207; doi:10.7554/eLife.76207)
Supplement: Figure 1—source data 1. [file elife-76207-fig1-data1.pdf]

Fig.1C

BV/TV (%)	ZOL	HMDP	Vehicle
	7.31	2.30	1.25
	5.49	1.74	1.68
	8.60	3.15	1.62
	4.87	1.83	4.91

ConnD (1/mr	ZOL	HMDP	Vehicle
	96.35	3.82	0.52
	65.61	4.91	2.60
	107.98	7.19	1.12
	62.13	0.55	54.19

Tb.N (1/mm)	ZOL	HMDP	Vehicle
	4.50	3.58	3.14
	4.00	3.53	3.57
	4.04	3.63	3.35
	3.74	3.52	4.44

Tb.Th (mm)	ZOL	HMDP	Vehicle
	0.037	0.029	0.032
	0.037	0.025	0.029
	0.042	0.032	0.025
	0.037	0.026	0.032

Tb.Sp (mm)	ZOL	HMDP	Vehicle
	0.22	0.29	0.32
	0.25	0.28	0.29
	0.25	0.28	0.30
	0.27	0.29	0.23
