# Supplementary material for: Mechanism of bisphosphonate-related osteonecrosis of the jaw (BRONJ) revealed by targeted removal of legacy bisphosphonate from jawbone using competing inert hydroxymethylene diphosphonate
Source: eLife. 2022 Aug 26;11:e76207. doi: 10.7554/eLife.76207 (PMC9489207; doi:10.7554/eLife.76207)
Supplement: Figure 1—source data 2. [file elife-76207-fig1-data2.pdf]

Fig.1D

	10 μM HMDP			Blank
	0x	1x	2x	
	1.60E+10	1.13E+10	6.89E+09	1.57E+09
	2.10E+10	1.09E+10	7.44E+09	1.66E+09
	2.13E+10	1.22E+10	6.45E+09	1.64E+09
