# Supplementary material for: Mechanism of bisphosphonate-related osteonecrosis of the jaw (BRONJ) revealed by targeted removal of legacy bisphosphonate from jawbone using competing inert hydroxymethylene diphosphonate
Source: eLife. 2022 Aug 26;11:e76207. doi: 10.7554/eLife.76207 (PMC9489207; doi:10.7554/eLife.76207)
Supplement: Figure 1—source data 3. [file elife-76207-fig1-data3.pdf]

Fig.1E

①			②		
Vehicle	HMDP 1 x	HMDP 2x	Vehicle	HMDP 1 x	HMDP 2x
2.40E+09	2.98E+09	3.32E+09	2.89E+08	2.69E+08	2.38E+08
2.51E+09	2.65E+09	2.84E+09	3.30E+08	2.83E+08	3.09E+08
3.55E+09	3.55E+09	3.18E+09	3.15E+08	3.40E+08	2.66E+08

③			④		
Vehicle	HMDP 1 x	HMDP 2x	Vehicle	HMDP 1 x	HMDP 2x
1.75E+08	1.60E+08	1.70E+08	1.36E+08	1.46E+08	1.47E+08
1.69E+08	1.43E+08	1.52E+08	1.34E+08	1.08E+08	1.21E+08
1.64E+08	1.60E+08	1.32E+08	1.36E+08	1.38E+08	1.19E+08

⑤			⑥		
Vehicle	HMDP 1 x	HMDP 2x	Vehicle	HMDP 1 x	HMDP 2x
1.57E+08	3.85E+08	4.97E+08	1.42E+08	1.72E+08	2.13E+08
1.09E+08	3.77E+08	3.92E+08	1.25E+08	2.27E+08	2.44E+08
1.92E+08	2.74E+08	4.36E+08	1.11E+08	2.15E+08	2.28E+08

⑦			⑧		
Vehicle	HMDP 1 x	HMDP 2x	Vehicle	HMDP 1 x	HMDP 2x
1.02E+08	1.89E+08	1.96E+08	1.21E+08	1.50E+08	1.83E+08
1.21E+08	1.12E+08	1.87E+08	1.06E+08	9.96E+07	1.55E+08
9.87E+07	1.03E+08	1.77E+08	8.55E+07	9.24E+07	1.42E+08

⑨			⑩		
Vehicle	HMDP 1 x	HMDP 2x	Vehicle	HMDP 1 x	HMDP 2x
9.80E+07	1.39E+08	3.21E+08	5.90E+07	9.43E+07	1.57E+08
9.40E+07	1.64E+08	3.09E+08	8.56E+07	1.06E+08	1.25E+08
9.89E+07	9.98E+07	1.78E+08	1.11E+08	1.23E+08	1.05E+08

⑪			⑫		
Vehicle	HMDP 1 x	HMDP 2x	Vehicle	HMDP 1 x	HMDP 2x
8.37E+07	8.67E+07	1.36E+08	4.02E+07	4.84E+07	1.26E+08
9.44E+07	9.38E+07	9.27E+07	7.15E+07	8.90E+07	9.01E+07
1.03E+08	9.37E+07	8.95E+07	9.03E+07	9.57E+07	1.01E+08
