# Supplementary material for: Mechanism of bisphosphonate-related osteonecrosis of the jaw (BRONJ) revealed by targeted removal of legacy bisphosphonate from jawbone using competing inert hydroxymethylene diphosphonate
Source: eLife. 2022 Aug 26;11:e76207. doi: 10.7554/eLife.76207 (PMC9489207; doi:10.7554/eLife.76207)
Supplement: Figure 1—source data 4. [file elife-76207-fig1-data4.pdf]

Fig.1F

	10 μM HMDP			Blank
	0x	1x	2x	
	1.92	5.02	16.35	14.10
	1.53	6.29	9.67	15.05
	0.93	5.19	11.21	15.63
