# Supplementary material for: Mechanism of bisphosphonate-related osteonecrosis of the jaw (BRONJ) revealed by targeted removal of legacy bisphosphonate from jawbone using competing inert hydroxymethylene diphosphonate
Source: eLife. 2022 Aug 26;11:e76207. doi: 10.7554/eLife.76207 (PMC9489207; doi:10.7554/eLife.76207)
Supplement: Figure 2—source data 1. [file elife-76207-fig2-data1.pdf]

Fig.2F

		AF647-ZOL-DNV		
	CTRL	25 µM	75 µM	200 µM
	44.981	52.945	50.049	60.843
	54.789	60.569	60.394	58.686
	39.503	59.033	70.389	64.529
	51.885	47.985	62.893	57.441
	40.859	39.689	49.9	53.983
		45.693	52.586	52.553
