# Supplementary material for: Mechanism of bisphosphonate-related osteonecrosis of the jaw (BRONJ) revealed by targeted removal of legacy bisphosphonate from jawbone using competing inert hydroxymethylene diphosphonate
Source: eLife. 2022 Aug 26;11:e76207. doi: 10.7554/eLife.76207 (PMC9489207; doi:10.7554/eLife.76207)
Supplement: Figure 2—source data 2. [file elife-76207-fig2-data2.pdf]

Fig.2G

	CTRL	AF647-ZOL-nDNV in MQW	AF647-ZOL-nDNV in PEG	AF647-ZOL-DNV in MQW	AF647-ZOL-DNV in PEG
	53.29705	57.83661	53.81623	75.295	54.439
	54.56349	58.91001	47.95108	76.978	55.604
	48.33326	57.22558	55.82214	61.764	84.594
	51.90918	55.22512	49.63551	69.914	65.185
	48.91939	47.55332	51.14558	76.731	54.374
	52.75763	57.63268	49.04705	68.142	57.87
	54.751	55.77855	64.48561	67.624	59.037
	51.553	51.89984	49.57013	54.127	52.335
	50.353				
	49.863				
