# Supplementary material for: Mechanism of bisphosphonate-related osteonecrosis of the jaw (BRONJ) revealed by targeted removal of legacy bisphosphonate from jawbone using competing inert hydroxymethylene diphosphonate
Source: eLife. 2022 Aug 26;11:e76207. doi: 10.7554/eLife.76207 (PMC9489207; doi:10.7554/eLife.76207)
Supplement: Figure 3—source data 1. [file elife-76207-fig3-data1.pdf]

Fig.3I

ZOL IV	-	-	+	+	+
Treatment	-	-	Empty-DNV	HMDP alone	HMDP-DNV
	19.69	91.52	50.58	1.03	57.77
	14.40	76.34	6.56	3.19	79.70
	7.13	47.94	18.16	45.08	35.28
			24.74	1.74	50.65
			33.83	21.03	88.97
			1.63	55.96	90.58
