# Supplementary material for: Mechanism of bisphosphonate-related osteonecrosis of the jaw (BRONJ) revealed by targeted removal of legacy bisphosphonate from jawbone using competing inert hydroxymethylene diphosphonate
Source: eLife. 2022 Aug 26;11:e76207. doi: 10.7554/eLife.76207 (PMC9489207; doi:10.7554/eLife.76207)
Supplement: Figure 3—source data 2. [file elife-76207-fig3-data2.pdf]

Fig.3J

ZOL IV	-	-	+	+	+
Intra-oral treatment	-	-	Empty-DNV	HMDP alone	HMDP-DNV
	24.20	20.81	26.67	53.00	22.69
	15.76	21.41	40.34	24.14	26.43
	15.34	10.97	34.42	38.01	24.18
			43.35	42.78	31.14
			40.46	40.39	15.45
			35.21	40.39	18.57
