# Supplementary material for: Mechanism of bisphosphonate-related osteonecrosis of the jaw (BRONJ) revealed by targeted removal of legacy bisphosphonate from jawbone using competing inert hydroxymethylene diphosphonate
Source: eLife. 2022 Aug 26;11:e76207. doi: 10.7554/eLife.76207 (PMC9489207; doi:10.7554/eLife.76207)
Supplement: Figure 4—source data 1. [file elife-76207-fig4-data1.pdf]

Fig.4C

	1 week	2 week		4 week	
Treatment	-	-	HMDP-DNV	-	HMDP-DNV
	41.24	29.26	1.52	58.37	2.17
	45.18	53.87	1.88	6.70	3.49
	46.69	13.23	5.94	45.88	0.66
	41.16	46.56	2.74	8.04	1.45
	56.65	49.62	0.99	15.50	3.22
		43.57	2.13	12.33	1.75
