# Supplementary material for: Mechanism of bisphosphonate-related osteonecrosis of the jaw (BRONJ) revealed by targeted removal of legacy bisphosphonate from jawbone using competing inert hydroxymethylene diphosphonate
Source: eLife. 2022 Aug 26;11:e76207. doi: 10.7554/eLife.76207 (PMC9489207; doi:10.7554/eLife.76207)
Supplement: Figure 4—source data 2. [file elife-76207-fig4-data2.pdf]

Fig.4D

	1 week	2 week		4 week	
Treatment	-	-	HMDP-DNV	-	HMDP-DNV
	234.34	300.43	95.79	281.07	44.12
	155.07	220.31	43.25	159.82	48.87
	251.39	71.89	39.12	139.36	1.38
	203.34	264.62	43.17	150.64	15.04
	111.39	201.76	88.95	196.45	49.13
		196.19	170.44	166.53	2.01
