# Supplementary material for: Mechanism of bisphosphonate-related osteonecrosis of the jaw (BRONJ) revealed by targeted removal of legacy bisphosphonate from jawbone using competing inert hydroxymethylene diphosphonate
Source: eLife. 2022 Aug 26;11:e76207. doi: 10.7554/eLife.76207 (PMC9489207; doi:10.7554/eLife.76207)
Supplement: Figure 4—source data 3. [file elife-76207-fig4-data3.pdf]

Fig.4E

	1 week	2 week		4 week	
Treatment	-	-	HMDP-DNV	-	HMDP-DNV
	5.86	9.01	63.42	41.96	47.09
	6.10	13.21	60.10	51.79	67.87
	1.24	12.28	53.16	61.63	54.57
	13.58	7.65	66.26	48.03	63.13
	12.61	24.07	51.97	62.51	65.21
		22.99	44.26	27.54	32.84
