# Supplementary material for: Mechanism of bisphosphonate-related osteonecrosis of the jaw (BRONJ) revealed by targeted removal of legacy bisphosphonate from jawbone using competing inert hydroxymethylene diphosphonate
Source: eLife. 2022 Aug 26;11:e76207. doi: 10.7554/eLife.76207 (PMC9489207; doi:10.7554/eLife.76207)
Supplement: Figure 4—source data 4. [file elife-76207-fig4-data4.pdf]

Fig.4F

	1 week	2 week		4 week	
Treatment	-	-	HMDP-DNV	-	HMDP-DNV
	25.43	44.50	32.83	55.92	4.44
	38.15	43.53	31.83	62.96	1.83
	49.70	42.19	18.71	60.81	3.75
	29.28	42.34	32.30	49.02	3.42
	31.50	42.56	28.19	80.95	3.35
		61.21	37.14	52.79	3.33
