# Supplementary material for: Mechanism of bisphosphonate-related osteonecrosis of the jaw (BRONJ) revealed by targeted removal of legacy bisphosphonate from jawbone using competing inert hydroxymethylene diphosphonate
Source: eLife. 2022 Aug 26;11:e76207. doi: 10.7554/eLife.76207 (PMC9489207; doi:10.7554/eLife.76207)
Supplement: Figure 4—source data 5. [file elife-76207-fig4-data5.pdf]

Fig.4G

Treatment	-	HMDP-DNV
	8.63	14.88
	16.28	5.75
	13.11	10.61
	11.26	12.82
	14.62	18.72
	12.71	15.03
