# Supplementary material for: Mechanism of bisphosphonate-related osteonecrosis of the jaw (BRONJ) revealed by targeted removal of legacy bisphosphonate from jawbone using competing inert hydroxymethylene diphosphonate
Source: eLife. 2022 Aug 26;11:e76207. doi: 10.7554/eLife.76207 (PMC9489207; doi:10.7554/eLife.76207)
Supplement: Figure 5—source data 1. [file elife-76207-fig5-data1.pdf]

Fig. 5D

	2 week		4 week	
Treatment	-	HMDP-DNV	-	HMDP-DNV
	9	10	9	0
	8	4	8	2
	13	4	15	1
	8	5	9	0
	8	6	8	1
