# Supplementary material for: Mechanism of bisphosphonate-related osteonecrosis of the jaw (BRONJ) revealed by targeted removal of legacy bisphosphonate from jawbone using competing inert hydroxymethylene diphosphonate
Source: eLife. 2022 Aug 26;11:e76207. doi: 10.7554/eLife.76207 (PMC9489207; doi:10.7554/eLife.76207)
Supplement: Figure 5—source data 2. [file elife-76207-fig5-data2.pdf]

Fig. 5E

Zone A	2 week		4 week	
Treatment	-	HMDP-DNV	-	HMDP-DNV
	6	8	5	0
	4	4	4	2
	8	4	12	1
	5	5	6	0
	3	6	4	0

Zone B	2 week		4 week	
Treatment	-	HMDP-DNV	-	HMDP-DNV
	3	2	4	0
	4	0	4	0
	5	0	3	0
	3	0	3	0
	5	0	4	1
